# Partially spatially coherent digital holographic microscopy and machine learning for quantitative analysis of human spermatozoa under oxidative stress condition

**DOI:** 10.1038/s41598-019-39523-5

**Published:** 2019-03-05

**Authors:** Vishesh Dubey, Daria Popova, Azeem Ahmad, Ganesh Acharya, Purusotam Basnet, Dalip Singh Mehta, Balpreet Singh Ahluwalia

**Affiliations:** 1 Applied Optics and Biophotonics Laboratory, Department of Physics, Indian Institute of Technology Delhi, Delhi, India; 2 Department of Physics and Technology, UiT The Arctic Univ. of Norway, Tromsø, Norway; 3 Department of Clinical Medicine, UiT The Arctic Univ. of Norway, Tromsø, Norway; 4 Department of Clinical Science, Intervention and Technology Karolinska Univ. Hospital, Karolinska, Sweden

## Abstract

Semen quality assessed by sperm count and sperm cell characteristics such as morphology and motility, is considered to be the main determinant of men’s reproductive health. Therefore, sperm cell selection is vital in assisted reproductive technology (ART) used for the treatment of infertility. Conventional bright field optical microscopy is widely utilized for the imaging and selection of sperm cells based on the qualitative analysis by experienced clinicians. In this study, we report the development of a highly sensitive quantitative phase microscopy (QPM) using partially spatially coherent light source, which is a label-free, non-invasive and high-resolution technique to quantify various biophysical parameters. The partial spatial coherence nature of light source provides a significant improvement in spatial phase sensitivity and hence reconstruction of the phase of the entire sperm cell is demonstrated, which was otherwise not possible using highly spatially coherent light source. High sensitivity of the system enables quantitative phase imaging of the specimens having very low refractive index contrast with respect to the medium like tail of the sperm cells. Further, it also benefits with accurate quantification of 3D-morphological parameters of sperm cells which might be helpful in the infertility treatment. The quantitative analysis of more than 2500 sperm cells under hydrogen peroxide (H_2_O_2_) induced oxidative stress condition is demonstrated. It is further correlated with motility of sperm cell to study the effect of oxidative stress on healthy sperm cells. The results exhibit a decrease in the maximum phase values of the sperm head as well as decrease in the sperm cell’s motility with increasing oxidative stress, i.e., H_2_O_2_ concentration. Various morphological and texture parameters were extracted from the phase maps and subsequently support vector machine (SVM) based machine learning algorithm is employed for the classification of the control and the stressed sperms cells. The algorithm achieves an area under the receiver operator characteristic (ROC) curve of 89.93% based on the all morphological and texture parameters with a sensitivity of 91.18%. The proposed approach can be implemented for live sperm cells selection in ART procedure for the treatment of infertility.

## Introduction

Infertility affects approximately 15% of couple worldwide^1^. Male factor infertility affects approximately 7% of the general male population, and poor semen quality is considered to be one of the main factors^2^. Along with inherited genetic problems, meiotic abnormalities causing miscarriages and inflammation, sperm abnormalities can be due to oxidative stress activated during the process of *in-vitro* fertilization (IVF) itself^3^. Standard sperm manipulations, such as wash from seminal plasma, cryopreservation and centrifugation, may impair antioxidant defence and increase the production of reactive oxygen species (ROS)^4,5^. Low level of ROS modulates signalling pathways required for human sperm activation, whereas high level impairs sperm function, leading to infertility. Specifically, oxidative stress is known to affect the integrity of the sperm genome, result in lipid peroxidation, loss in membrane fluidity, and decrease in sperm motility^6,7^.

Adverse effects of oxidative stress might not be explored with light microscopy. For that reason, sperm cells with impaired fertilizing potential can be picked by embryologists for intracytoplasmic sperm injection (ICSI). At the same time, routine oxidative stress screening is not performed in IVF laboratories because of high cost and complexity of standard tests^8^. Moreover, implementation of rapid diagnostics could replace long and cumbersome multi-step analytic procedures that require complex experimental equipment.

The human sperm cells are relatively transparent in nature and have almost similar optical properties as surroundings leading to low refractive index (RI) contrast. Therefore, it is difficult to obtain a good contrast image by using bright field microscope. Several optical techniques have been developed for the contrast enhancement of sample images^9–11^, however, they do not provide any quantitative information of the specimen^12,13^. When the light passes through the specimen, the optical path delay (OPD) is generated in light field due to the RI difference between the cell and the surrounding medium. The OPD is measured using quantitative phase microscopy (QPM) techniques, which are based on the principle of interferometry. It can be further utilized to measure several optical properties of specimen. The QPM techniques have been employed for the visualization and the evaluation of specimens that are particularly useful in cell biology^12–14^. The key advantage of these techniques is that they provide high resolution 3-D quantitative information of the specimen without any labelling.

In this study, we have investigated the effects of externally induced oxidative stress by treating healthy sperm cells with hydrogen peroxide (H_2_O_2_) using spatially low coherent QPM and further the findings are correlated with clinically relevant motility parameter of the sperm cells. A number of studies have been implemented for the quantitative assessment of normal and immotile sperm cells utilizing QPM^15–19^, however the effect of oxidative stress on the morphology and motility of the sperm cells is not done previously. In addition, existing QPM either utilized a narrowband (i.e., lasers) or broadband (i.e., light emitting diodes and halogen lamp) light sources for phase imaging of sperm cells^17–20^. The use of highly temporal and spatial coherent light sources, like lasers, degrades the interferogram’s quality due to speckle and spurious fringe formation, which eventually reduces the spatial sensitivity of the system^21–23^. This makes difficult to perform quantitative phase imaging (QPI) of the tail of sperm cells as it offers minute OPD^18,19^. The phase sensitivity can be improved by utilizing broadband light sources like white light, light emitting diodes and super-luminescent diodes^24–26^. However, such light sources require chromatic aberration corrected optics and dispersion compensation mechanism. In addition, single shot phase recovery over the whole camera field of view (FOV) is not possible due to low fringe density with low temporal coherent light sources^25^. Thus, a monochromatic extended (i.e., pseudo-thermal) light source can be implemented in QPM technique, which carries advantages of both narrow-band and broad-band light sources. Several methods have been proposed to synthesize pseudo-thermal light sources using rotating diffuser and vibrating multiple mode fiber bundle (MMFB), previously^22,23^.

A monochromatic laser beam is passed through a rotating diffuse to synthesize a pseudo-thermal light source which carries high temporal coherence (helps to obtain high fringe density over whole camera FOV) and low spatial coherence (generate speckle and spurious fringe free interfergrams) properties. Such light source is employed with Linnik-type interference microscopy system to record off-axis holograms of sperm cells. The phase maps of sperm cells are then recovered with improved spatial phase sensitivity of the order of 20 ± 1.5 mrad. It is exhibited that phase map of the tail of sperm cell is nicely recovered with pseudo-thermal light source, which is otherwise not possible in laser based phase imaging. Most of the QPM techniques are, therefore, implemented only on dried sperm cells to recover phase map of the tail of sperm cells, previously^17,19^. Further, diagnosis of both the head and the tail of sperm cells are important for the procedures of artificial reproductive technologies. According to the WHO criteria, healthy tail having principle piece should be uniform along its length, be thinner than the midpiece, with a length of about 45 µm and without any sharp angle^27^. Thus, quantitative assessment of sperm tail can help to choose a healthy sperm in the clinical practice. QPM may also provide a better visualization to detect the abnormalities like defects of head neck attachment, primary ciliary dyskinesia (PCD), or dysplasia of fibrosis sheath (DFS).

It was observed that the optical thickness of the sperm’s head decreases as a function of increase in the H_2_O_2_ concentration. Several morphological and texture parameters were extracted from the phase maps to measure the changes during oxidative stress. A support vector machine (SVM) based classifier is developed for the classification of normal and stressed sperm cells. The morphological and texture parameters extracted from phase maps were used to train the algorithm for better classification. For the training of the classifier, 60% of total samples were used and rest 40% were used as test specimen. We have achieved an accuracy of 89.93% for the classification of control and test sperm cells with SVM model. The observations support the hypothesis that changes caused by the oxidative stress could result in the decrease of maximum phase value of the sperm cell as compared to the normal one. The findings of QPM were correlated with a dose-dependent decrease in progressive motility of the sperm cells. The decrease in sperm motility with an increase in the H_2_O_2_ concentration was observed as compared to the controlled samples.

## Methods and Materials

### Principle of DHM

DHM is based on the principle of interferometry, in which a full or partially coherent light is divided into two beams, one as reference and other illuminates the specimen called object wave. Further, the scattered wave from object and reference waves interfere to generate the hologram and the 2D intensity distribution can be expressed as:

1 $$h(x,y)=a(x,y)+b(x,y)cos[2\pi i({f}_{x}x+{f}_{y}y)+{\rm{\Delta }}\varphi (x,y)]$$

where a(x, y) and b(x, y) represent the background (DC) and the modulation terms, respectively, Δ*ϕ*(*x*, *y*) is the phase difference between the object and reference fields, *f*_*x*_ and *f*_*y*_ are the spatial frequencies of the interference pattern along x and y directions, respectively.

For the convenience, the above intensity pattern of hologram can be rewritten in the following form

$$h(x,y)=a(x,y)+c(x,y)exp[2\pi i({f}_{x}x+{f}_{y}y)]+{c}^{\ast }(x,y)exp[\,-\,2\pi i({f}_{x}x+{f}_{y}y)]$$

where

$$c(x,y)=b(x,y)exp(i\varphi (x,y))$$

The hologram reconstruction allows the retrieval of the complex object field. To retrieve the phase information, Fourier transform of the hologram is taken and one of the twin image peaks is filtered with numeric band pass filter in the frequency domain. Further, inverse Fourier transform is performed to reconstruct the hologram (*h*_*filt*_) as a 2D array of complex numbers. The phase profile of the specimen is then simply measured as:

2 $${\rm{\Delta }}\varphi ={\arctan }(\frac{imag({h}_{filt})}{real({h}_{filt})})$$

The phase Δ*ϕ* depends on the thickness of the specimen and the RI difference of the specimen and the media containing the object itself. This phase variation having information of the morphology of specimen under investigation thus holography provides a 3D topographic profile of the specimen. The phase is related to the optical path difference (OPD) by the relation^12^:

3 $$\varphi (x,y)=\frac{2\pi }{\lambda }\times 2h(x,y)\ast \{{n}_{s}(x,y)-{n}_{0}(x,y)\}$$

where λ is the wavelength of incident light, *h* is the geometrical thickness of the specimen; *n*_*s*_ and *n*_*o*_ are the refractive indices of the specimen and surrounding medium, respectively and there is an extra factor of 2 appears because the reflection configuration is utilized to record the hologram.

### Morphological and Statistical Analysis

The analysis of recovered phase is very important for the image based computer-aided diagnosis (CAD), which provides excellent accuracy in early stage disease detection^28,29^. Machine learning is a subfield of computer science having a range of applications in biomedical imaging, which uses the extracted morphological and texture features of the image to make predictions^28,29^. For the classification of sperm cells under control and oxidative stress conditions, the phase map of the head of sperm cells are utilized for the calculation of the various texture parameters, which were further utilized in SVM algorithm.

Once the phase maps of the sperm cells were extracted from the hologram, the head of the sperm cell isolated to extract the phase map based morphological and texture features. The optical thickness (OT) is related to the phase of the specimen by the relation $OT=\varphi (x,y)\ast \lambda /4\pi$ (for reflection geometry), where λ is the wavelength of the light. The measured OT is utilized to measure the volume of sperm head and can be calculated by integrating OT over projected area as^30,31^

4 $$V={\iint }^{}OT(x,y){dxdy}$$

where *dx* and *dy* are the calibrated pixel width along x and y directions, respectively.

The area element *dS* of the cell surface is calculated by Monge parameterization defined as^30,32^

5 $$dS={dxdy}\sqrt{1+{G}_{x}^{2}+{G}_{y}^{2}}$$

where *G*_*x*_ and *G*_*y*_ are the gradients along the *x* and *y* directions, respectively. Further, the surface area ‘*S*’ is defined as the sum of all the area elements and the projected area^32^. Next, sphericity ‘$\Psi$’ of the sperm head was determined, whose values lie between 0 and 1 (for laminar disk and perfect sphere, respectively). It is defined as the ratio between the surface area (*S*) of a cell with the volume of the same cell and calculated as^30,31,33^

6 $${\Psi }=\frac{4.84\ast {V}^{2/3}}{S}$$

### Semen preparation

Semen samples were obtained from men who attended the IVF clinic for the investigation and/treatment of infertility. The Regional Committee for Medical and Health Research Ethics of Norway (REK_nord) approved the project. An informed consent was obtained from all participants.

The semen sample was collected according to the guidelines of the World Health Organization with an abstinence period of 3–5 days. After collection, the sample was allowed to liquefy for 30–40 min. Sperm counts were evaluated using the Neubauer-improved counting chambers. All ejaculates used in the experiments had an original sperm concentration more than 60 million of cells per milliliter, progressive motility more than 50% and with normal morphology >14% following strict criteria. The sperm fraction with high motility was isolated by density gradient centrifugation method (Vitrolife, Sweeden). One milliliter of semen was carefully placed on the gradient layers (90% and 45% layers) and centrifuged at 500 *g* for 20 min. The pellet from the centrifuge tube was washed twice with human Quinn’s sperm washing medium (SM; Origio, Denmark) at 300 *g* for 10 min. The supernatant was discarded, and the pellet was re-suspended in QA fert-medium supplemented with 5 mg/ml HSA and was used for following procedures.

To perform oxidative stress experiment, sperm sample was diluted to a concentration of 5.0 × 10^6^cells/ml using culture medium. Further, 96 well tissue culture plates were filled with sperm in medium (Quinn’s Advantage Protein plus Fertilization medium, SAGE, Denmark) with different concentrations of H_2_O_2_ (10 µM, 40 µM, 70 µM, 100 µM) and the reference chamber was filled with the same concentration of semen without H_2_O_2_. The samples were incubated for 1 hour at 37 °C, 5–6% CO_2_. After incubation motility of each sperm sample was graded in two clusters: progressive motility (PR) and non-progressive motility (NP), which were reported as in percentages.

For QPM, the cells of each concentration were placed in a PDMS chamber on reflecting silicon (Si) chip after 1 hour of incubation. To immobilize the sperm cells, samples were fixed with 4% PFA for 30 min at RT and washed in phosphate-buffered saline (PBS) for 5 min. Finally, 50 μL of PBS were added in the PDMS chamber with fixed cells and the samples were covered by cover glass.

### Experimental Details

**Figure 1:**
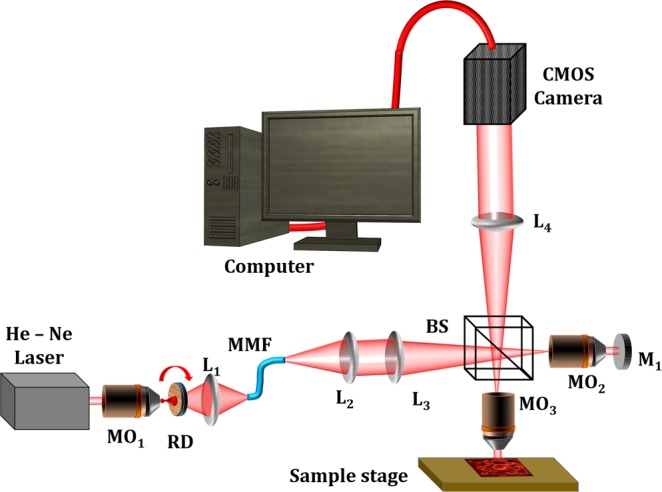
Schematic diagram of the DHM setup with pseudo thermal light source for the acquiring the quantitative phase maps of sperm sample. (RD- rotating diffuser, L- lens, BS- Beam splitter, MO-microscope objective, MMFB- multiple multi-mode fiber bundle).

The schematic of the partial spatial coherence gated QPM/DHM system based on Linnik interferometer is shown in Fig. 1. To reduce the phase noise of the system, the spatial coherence of the laser light source is reduced and the resulting light beam illuminates the specimen. It is demonstrated that when a coherent light incident on a rotating diffuser (RD) and the diffused light is coupled into the  multiple multi-mode fiber bundle (MMFB)  then its output acts as a pseudo thermal light source having partial spatial and highly temporal coherence properties. The detailed study of the speckle reduction can be found elsewhere^21–23^.

A highly coherent laser light (He-Ne @632.8 nm) beam is expanded using microscope objective MO_1_ and passed through a RD. The beam spot size of 4.5 mm is made onto the diffuser plane to match the diameter of MMFB. The scattered photons are collected by lens L_1_ (focal length *f*_1_ = 50 mm) and pumped into the MMFB. The light from MMFB output is first collimated and then focused at the back-focal plane of the MO_3_ by utilizing the lenses L_2_ (*f*_2_ = 75 mm), L_3_ (*f*_3_ = 150 mm) and beam splitter (BS). Thus the samples are illuminated by a nearly collimated beam for their accurate phase imaging. In the reference arm an optically flat mirror (of the order of λ/10) is used. The reflected light from the reference mirror and the specimen are re-combined at BS to form interference pattern. The interferograms are then projected on the CMOS image sensor (Hamamatsu ORCA-Flash4.0 LT, C11440-42U) using tube lens L_4_ (*f*_4_ = 200 mm). The camera exposure time is kept 50 ms.

### Comparison of coherent laser and pseudo-thermal light source based phase imaging

**Figure 2:**
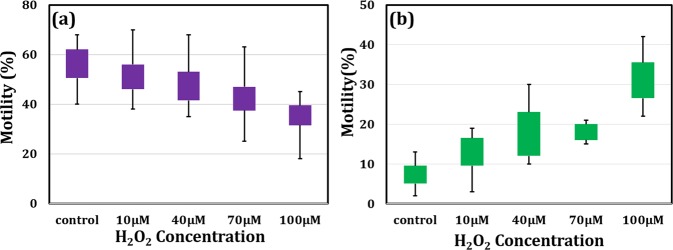
Measurement of the spatial phase sensitivity of QPM for direct laser and pseudo-thermal light sources. (**a**,**d**) are the interferograms obtained with healthy sperm cell as a test specimen, (**b**,**e**) reconstructed phase map of the sperm cell corresponding to (**a**,**d**), respectively and (**c**,**f**) spatial phase noise of the experimental setup for laser and pseudo-thermal light sources, respectively. Note that the scale of the color bars used in (**c**,**f**) having different values.

In the proposed geometry, a pseudo thermal light source is used to reduce the spatial phase noise of the system which further enhances the measurement accuracy of the system. First, we have compared the spatial phase sensitivity of the system by imaging the sperm cell with fully coherent and partially coherent (pseudo-thermal) light sources. Figure 2 shows interferogram, reconstructed phase map of a sperm cell and the spatial phase noise of the system for fully and partially spatially coherence light sources. The spatial phase sensitivity of the system is enhanced when the test specimen is illuminated by the partially spatially coherent light source. Figure 2(a,d) show the interferograms of the sperm cell utilizing direct laser and synthesized pseudo-thermal light sources, respectively. Highly coherent nature of light source leads to speckle and non-uniform illumination of the specimen as shown in Fig. 2(a), while pseudo-thermal light source provides a speckle free uniform illumination (Fig. 2(d)). The object is clearly visible in Fig. 2(d) with illumination of pseudo-thermal light source which is otherwise not visible with direct laser source (Fig. 2(a)). Figure 2(b,e) show their corresponding reconstructed phase maps of the interferograms depicted in Figs 2(a) and 2(d). It can be observed from the phase images that the finer features of the sperm cells i.e. neck and tail is not resolved in phase map of hologram recorded by the direct laser, while whole sperm cell is clearly reconstructed in case of pseudo-thermal light source. In case of direct laser, the generation of speckle and non-uniform illumination reduces the over-all spatial phase sensitivity of the system which results in poor resolution. The spatial phase sensitivity of the system for both kind of light sources were measured and compared. High spatial phase sensitivity is essential where minute phase variations in the target are needed to be quantified. Here, we utilized pseudo-thermal light source to enhance the spatial phase sensitivity of the phase microscopy system. The difference in phase values of the controlled and the 10 μM sperm cells is only 8%, which would be difficult to differentiate with direct laser based QPM technique due to high spatial phase noise. Figure 2(c,f) show the spatial phase noise of the system for the direct laser and pseudo-thermal light sources, respectively, where the color bars having different scale values. By measuring the standard deviation of the phase distribution, one can estimate the spatial phase sensitivity of the system. In our case, the phase sensitivity is observed to be 300 ± 11.9 mrad and 20 ± 1.5 mrad for direct laser and pseudo-thermal light source, respectively.

## Results and Discussion

### Sperm cell motility after treatment with different concentrations of H_2_O_2_

**Figure 3:**
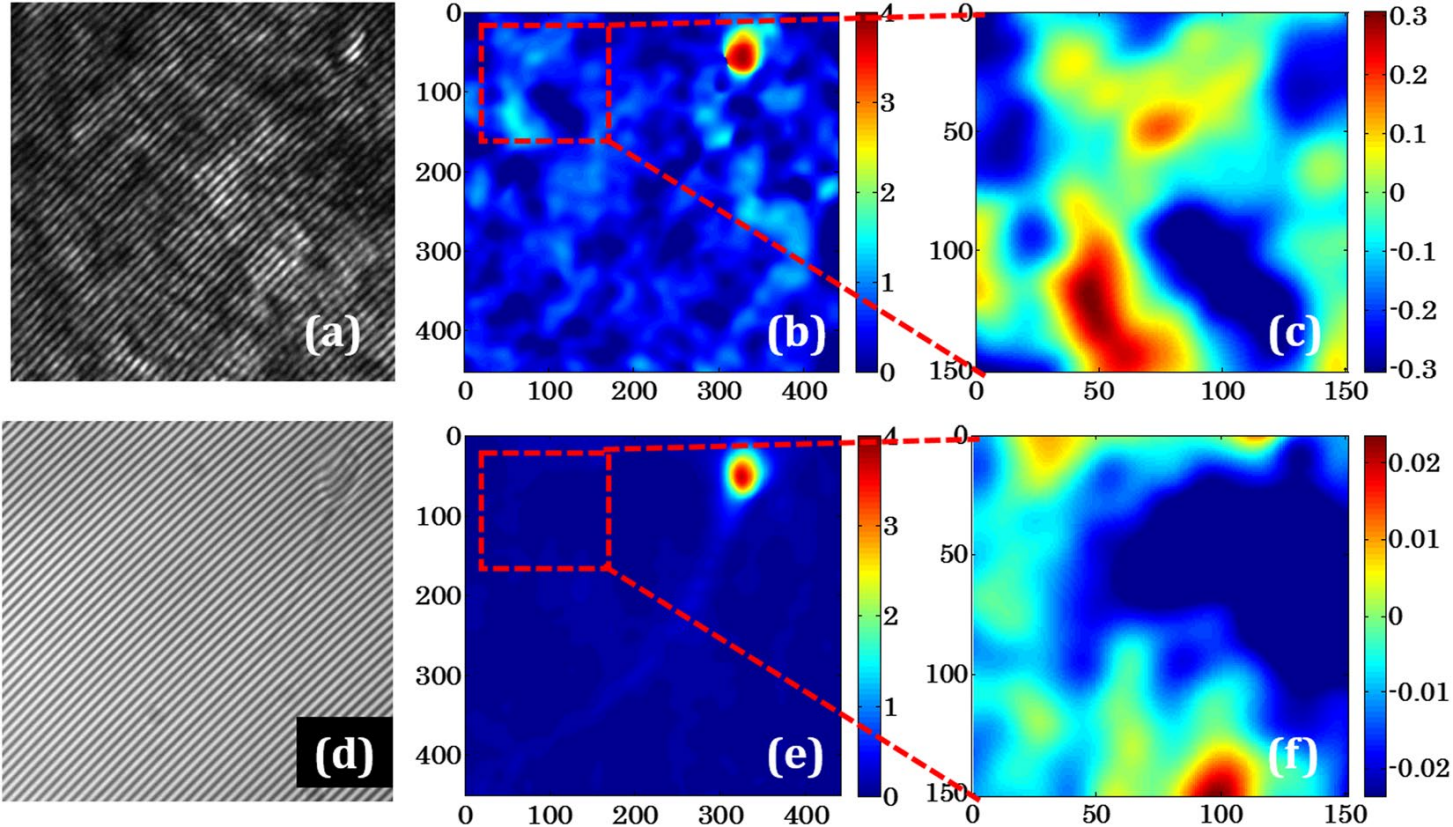
Effects of the H_2_O_2_ on the motility of sperm cells (**a**) changes in the percentage of progressive motility and (**b**) non-progressive motility of sperm cells after H_2_O_2_ treatment at different concentrations comparing to control (mean ± SE, p < 0.05 vs. control).

Significant differences in the motility parameters were detected when comparing the control sample with exposed samples with various H_2_O_2_ concentrations. The effect of different concentrations of H_2_O_2_ on progressive and non-progressive motility of spermatozoa are shown in Fig. 3(a,b) respectively (n = 7; seven ejaculates from different donors). Four different concentrations of H_2_O_2_ were tested (10 µM, 40 µM, 70 µM, 100 µM) for the oxidative stress study on sperm cells. Sperm samples were incubated for 1 hour at 37 °C in the absence (control) or presence (test) of H_2_O_2_.

Evaluation of cell motility was performed by experienced biologist according to WHO 2010 criteria^27^. The motility of progressive class was graded as moving actively, linearly or in a large circle, regardless of speed. Non-progressive motility was estimated as nonlinear movement with flagellar force hardly displacing the head, or with only flagellar or head trembling. At least 100 cells per each H_2_O_2_ concentration were analyzed. For the estimation of motility of sperm cells, the cells were kept in 96 well tissue culture plates and observed under the inverted microscope with 40X magnification objective lens. H_2_O_2_ produces a concentration-dependent decrease in progressive motility of sperm cells (ANOVA: p < 0.05). The concentration at 100 µM of H_2_O_2_ had the most significant effect on the decrease in numbers of progressive motile cells in comparison with other doses (Fig. 3(a), (10 µM: 51 ± 10.7; 40 µM: 48 ± 10.9; 70 µM: 42 ± 11.9; 100: µM 34 ± 8.8; versus control: 59 ± 10.8%, paired *t*-test, p < 0.05). At the same time H_2_O_2_ affects most on the non-progressive motility at the concentrations of 70 µM and 100 µM as compared with control (70 µM: 19 ± 3.9; 100 µM: 29 ± 7.4; versus control: 6 ± 6.6%, paired *t*-test, p < 0.05). Both doses 10 µM and 40 µM of H_2_O_2_ did not influence to the non-progressive motility (10 µM: 14 ± 4.8; 40 µM: 16 ± 5.4; versus control: 6 ± 6.6%, paired *t*-test, p > 0.05, (Fig. 3(b)). Effects on non-progressive motility were statistically significant at H_2_O_2_ concentration of 70 µM and 100 µM (p < 0.05 when compared with controls).

The effect of oxidative stress on sperm motility has been demonstrated in number of studies^34–36^. H_2_O_2_ is externally supplemented agent to induce oxidative stress on sperm cells. Our results support the previous studies that the extent of motility decrease depends on the concentration of H_2_O_2_. The underlying mechanism of H_2_O_2_ influence to sperm motility is described previously^37,38^. Membrane lipids of sperm cells contain unsaturated fatty acids which are vulnerable to peroxidation. Sperm incubation with H_2_O_2_ triggers lipid peroxidation cascade results in membrane loss of flexibility and plasticity which determines disrupted tail motion^3,34,39^. Moreover, motility may be decreased because of restriction of energy production by damaged mitochondria after oxidation^40,41^.

### Quantitative phase imaging of sperm cells

**Figure 4:**
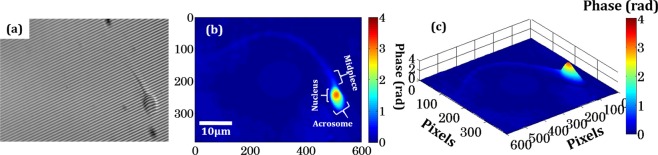
Digital holographic process and reconstructed phase maps of sperm cell (**a**) Typical hologram of the sperm cell recorded from partially coherent DHM setup, (**b**) reconstructed phase map of sperm cell with it’s basic structure and (**c**) pseudo 3D phase map of the sperm cell. (color bar is showing the phase in radian, blue for zero and deep red for maximum phase).

The quantitative morphological analysis of the sperm cells provides a better understanding of the behaviour of sperm cells under control and oxidative stress conditions. Figure 4 shows the recorded hologram and pseudo colour unwrapped phase map of sperm cell. Figure 4(a) shows a typical low spatial coherence hologram of the sperm cell and 2D view of the recovered phase map is shown in Fig. 4(b). The basic structure of the sperm cell composed of the head, mid piece, tail and end piece, the head is partially covered with nucleus and acrosome. Figure 4(c) shows the pseudo 3D phase map of the same sperm cell where maximum optical path delay is generated by the head of the sperm cell having value approximately 4 rad.

**Figure 5:**
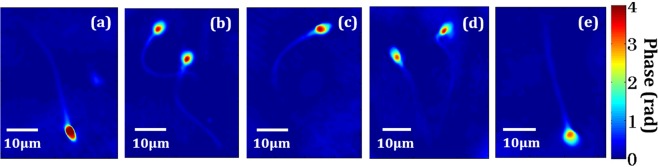
Pseudo-color plot of reconstructed phase maps of (**a**) normal sperm cells, and at different concentrations of (**b**) 10 μM, (**c**) 40 μM, (**d**) 70 μM and (**e**) 100 μM of concentration of H_2_O_2_, respectively (color bar shows the phase in radian, blue for zero and deep red for maximum phase).

**Figure 6:**
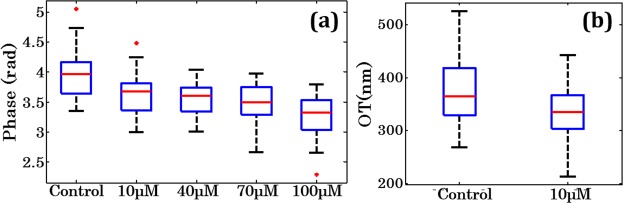
Whisker box plot for (**a**) maximum phase of the sperm head for control, 10 μM, 40 μM, 70 μM and 100 μM concentration of H_2_O_2_, respectively, and (**b**) optical thickness (OT) of the control vs. 10 μM concentration of H_2_O_2_. The central red lines indicate the median, and bottom and top sides of blue box indicate the 25^th^ and 75^th^ percentiles, respectively. The black lines extended vertically from blue boxes specify extreme data points without outliers, and ‘+’ symbols in red color are plotted for outliers.

The low spatial coherence QPM/DHM is further used for the evaluation of the effects of oxidative stress on the morphology of the human sperm cells. Figure 5 shows the recovered phase maps of sperm cells treated with different concentration of H_2_O_2_. Figure 5(a–e) show the reconstructed 3D phase maps for the control, 10 μM, 40 μM, 70 μM and 100 μM concentration of H_2_O_2_, respectively. It is observed from the phase images that with increasing concentration of H_2_O_2_, the maximum value of the phase of sperm head decreases which indicates that there is a change in the morphology of the sperm head. For the study of morphological changes in the sperm head during oxidative stress, several morphological parameters have been extracted from the phase maps. In total, more than 2500 sperm cells were analysed to measure the optical and morphological parameters. Figure 6(a) shows the whisker box plots of maximum phase of the sperm head at different concentrations of H_2_O_2_. Figure 6(b) shows the whisker box plot for the optical thickness of the sperm head for control and 10 μM concentration of the H_2_O_2_. The optical thickness decreases after oxidative stress which further changes the morphology of the sperm cells. The structure of sperm suggest that the nucleus is tallest part sperm followed by acrosome and mid-piece which allows observer to distinguish nucleus from acrosome^17,18^. The reconstructed phase map show a clearly detectable edge of cell boundary and maximum phase in the nucleus region. The identification and quantification of the optical thickness of nucleus may provide the deformation of nucleus during oxidative stress as shown in Fig. 5. The acrosome having significant low OT due to lesser thickness as compared to nucleus. Hence, the quantification of the change in the OT of nucleus during deformation can be a good marker for the quantification of oxidative stress. Here, we have chosen control and 10 μM concentration of the H_2_O_2_ only for the comparative study because there is almost linear decrease in the maximum phase with increasing concentration of the H_2_O_2_ (Fig. 6a).

### Characterization of morphological and texture parameters during oxidative stress

**Figure 7:**
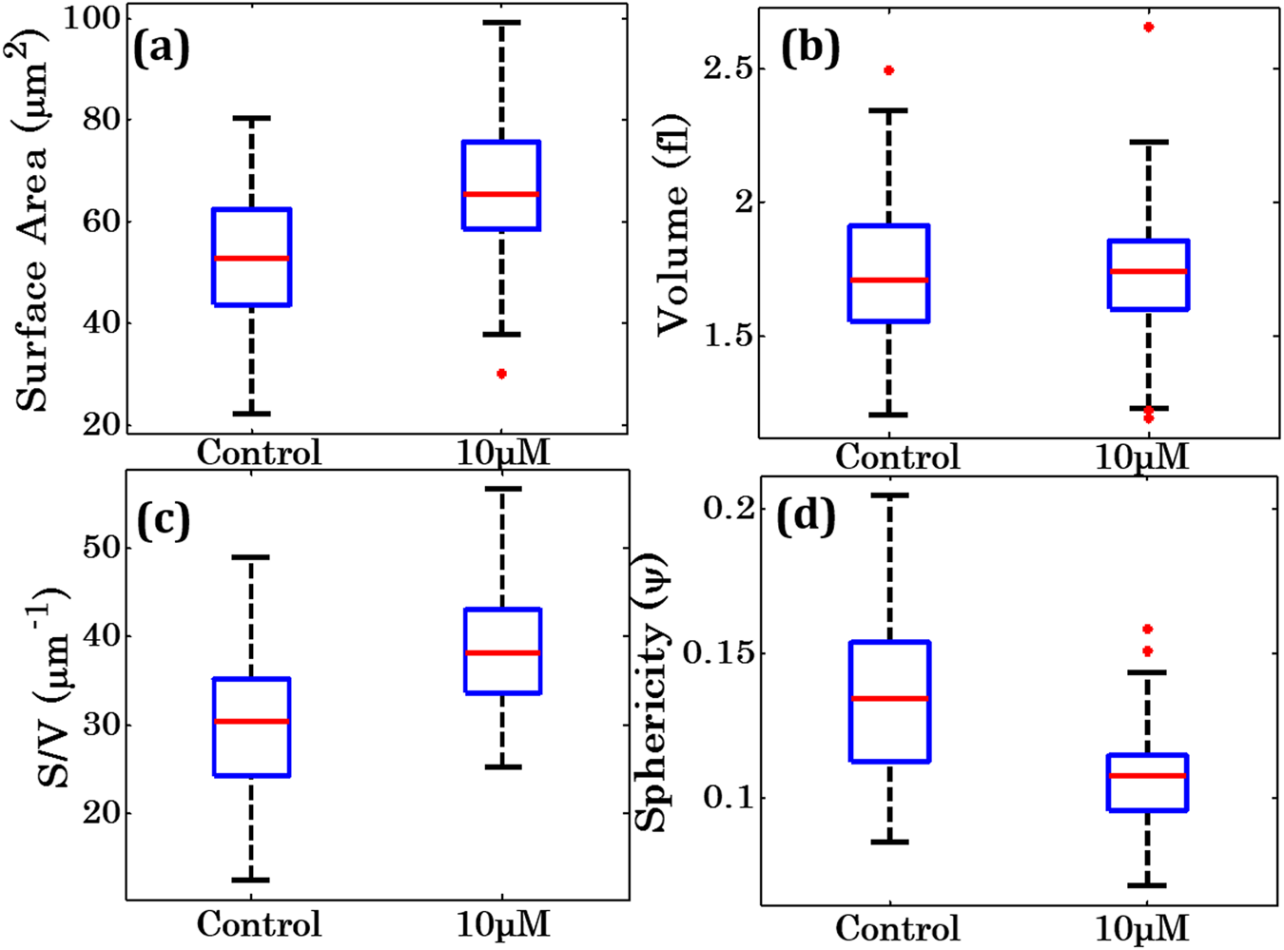
Morphological parameters of sperm head as expressed with the whisker box plots of (**a–d**) surface area, volume, surface area to volume ratio (S/V), and sphericity under normal (control) and oxidative stress conditions.

In order to determine the effects of oxidative stress on the morphology of the sperm cell head, the morphology of head is quantified from the phase maps using calculations describes in materials and method section. Surface area (S), volume (V), surface to volume ratio (S/V) and sphericity (Ψ) parameters were analysed for classification of control and 10 μM concentration of the H_2_O_2_. Figure 7(a–d) show the whisker box plots of these parameters for sperm head under control and oxidative stress conditions. The results show that the surface area increases in sperm cell head after the externally induced oxidative stress (Fig. 7a), while the volume is approximately constant during this process as can be seen in Fig. 7(b). There is an increase in the surface to volume ratio while sphericity decreases after oxidative stress. The increase in the S and S/V with decrease in ψ indicates that the flattening of the cells under stress assuming constant RI of the sperm head during whole process (Fig. 7(c,d)).

For the statistical analysis, selection and extraction of texture features are also important for the classification of any disease. Here, we have extracted various texture features from the phase maps of the sperm heads such as: mean, variance, entropy, kurtosis, skewness and energy. All the parameters were extracted by choosing a region of interest (ROI) of the sperm cells and listed in Table 1. There is a decrease in the mean value of the phase distribution over entire sperm head reflects the flattening of the sperm head i.e. decrease in the optical thickness of the sperm head after introducing 10 μM of H_2_O_2_ concentration. The decrease in the variance shows the less spread of data points around its mean value, while there is an increase in entropy predicts the increase in the randomness of phase distribution over entire sperm head. The increase in kurtosis and skewness show the more flatness and asymmetricity in phase distribution of sperm head. The decrease in the energy value shows the increase of heterogeneity in phase distribution of sperm cell head.

**Table 1:** Texture parameters of sperm cell head for normal (control) and externally induced oxidative stress (10 μM H_2_O_2_ concentration) conditions.

Texture Parameter	Definition	Median value of the parameter for	
		Control samples	10 μM concentration
Mean (μ)	$\frac{1}{N}\sum _{i=1}^{N}{\varphi }_{i}$	1.245	1.102
Variance (*σ*^2^)	$\frac{1}{N}\sum _{i=1}^{N}{({\varphi }_{i}-\mu )}^{2}$	0.965	0.857
Kurtosis	$\frac{1}{N}\sum _{i=1}^{N}{[\frac{({\varphi }_{i}-\mu )}{\sigma }]}^{4}-3$	2.197	2.469
Skewness	$\frac{1}{N}\sum _{i=1}^{N}{[\frac{({\varphi }_{i}-\mu )}{\sigma }]}^{3}$	0.669	0.772
Entropy	$-\sum _{i=1}^{N}p({x}_{i})lo{g}_{2}p({\varphi }_{i})$	5.056	5.448
Energy	$\sum _{i=1}^{N-1}\sum _{j=1}^{N-1}{({p}_{ij})}^{2}$	0.399	0.34

**Figure 8:**
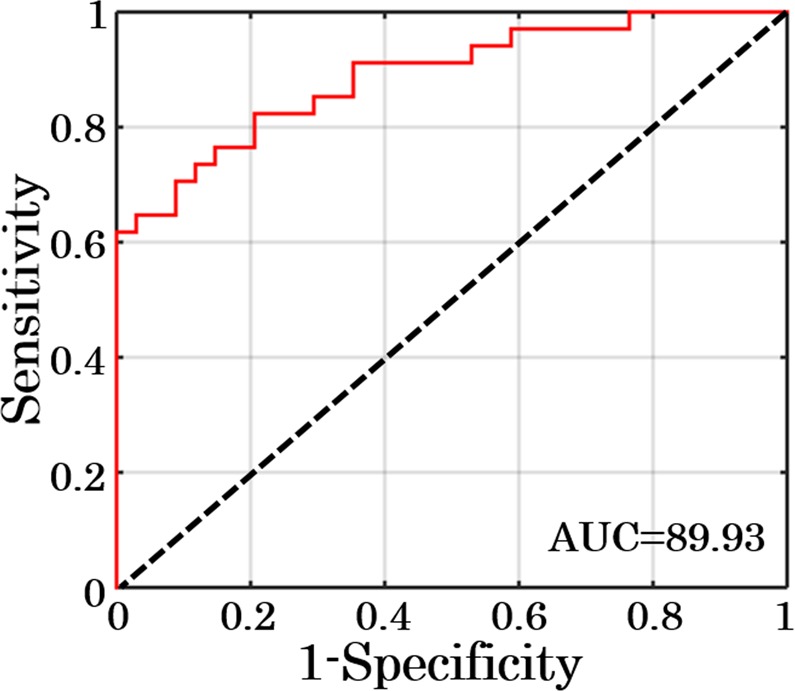
ROC curve for testing dataset of sperm head for control and 10 μM/ml H_2_O_2_ concentration treated sperm cells using eleven different morphological and texture parameters.

Once all the morphological and texture parameters were extracted from the phase maps of sperm cells, a support vector machine (SVM) classifier has been developed for the classification of the control and oxidative stress induced sperm cells^28,29,42^. Eleven parameters: OT, S, V, S/V, ψ, mean, variance, entropy, kurtosis, skewness and energy were utilized as input predictor variables and the genuine state of the sperm as a response variable i.e. 0 for control and 1 for 10 μM H_2_O_2_ concentration treated sperm cells. Sensitivity, specificity and area under receiver operating characteristic (ROC) curve were calculated to check the accuracy of the model. Total data points are divided into two sets, 60% for the training of the model and 40% for the testing purpose. Figure 8 shows the ROC curve for the testing data points with a specificity and sensitivity of 88.61% and 91.18%, respectively with an accuracy of 89.93% for the classification of control and test sperm cells.

## Conclusion

In present study, the capability of DHM using low spatial coherence light source alongwith SVM classifier exploited to measure change in morphology of sperm head after oxidative stress. It is exhibited that pseudo-thermal light source based phase imaging provides reconstruction of the biological structure having minute optical thickness (i.e., tail of the sperm cells), which is otherwise not possible under coherent illumination. It is known that fertilization capability of sperm cell is impaired under biological oxidative stress. The oxidative stress was induced using H_2_O_2_ treatment. Concentrations of H_2_O_2_ exceeding physiological threshold trigger the changes in semen leading to sperm cell dysfunction^7,35,38^. The evidence from previous studies suggests decrease of sperm cell motility due to membrane translocations of phospholipids^3,43^. In addition to membrane peroxidation, H_2_O_2_ initiates concentration-dependent increase of DNA fragmentation because of DNA strand breaks^3,36,44,45^. Using conventional microscopy, sperm cells with this type of anomalies might be amongst selected cells for intracytoplasmic sperm cell injection (ICSI) procedure leading to treatment failure. Therefore, it is of great significance to develop noninvasive methods for sperm cells selection. DHM appears to be one of the most promising noninvasive technique for the quantification of optical parameters of sperm cells^13,15,17^.

We found that H_2_O_2_ induces oxidative stress to the sperm cells which leads to the sperm cell dysfunction by decreasing its motility. The result of our study suggests the association between gradual progressive motility loss (Fig. 3) and the shift of optical properties of the sperm head (Figs 6, 7) after exposure to various concentrations of H_2_O_2_. The head morphology changes resulting from peroxidation might be due to de-condensation of genetic material because of DNA fragmentation. Quantitative evaluation of the phase shift by DHM provides an opportunity to use SVM to obtain new information on the exact structure and better distinguish sperm cells that are normal from those under oxidative stress (Fig. 8). Development of such machine learning algorithms could play an important role in automatic classification of the healthy and stressed sperm cells. The origin of decrease in the maximum phase of sperm head could be due to various reasons such as: deformation in nucleus, structural organization of sperm DNA, condensation of chromatin etc^19^. The morphometric values obtained in our study can provide the volumetric estimation for the quantitative comparison between control and H_2_O_2_ treated sperm cells. The correlation of decrease in the phase and deformation in the nucleus can be quantify by multimodal imaging in future where the boundary can be located by fluorescence imaging and QPM can provide the changes in the maximum phase of nucleus. QPM may have capability to quantify the changes due to fragmentation in DNA after introducing oxidative stress in human sperm which can be the motivation for this kind of analysis on the fertilization capacitance of sperm cell^46,47^.

One of the obstacles in IVF treatments is to recognize the sperm cells morphology by observing them under optical microscope whether it is under oxidative stress or not. However, by utilizing low spatial coherence DHM together with machine learning algorithms might provide better sperm selection during ICSI procedure. Moreover, as mentioned above, “hand-picked” spermatozoon for ICSI procedure might contain fragmented DNA, which can be detected indirectly by measuring sperm optical features using noninvasive, label-free QPM/DHM technique. We believe that our approach with DHM and machine learning based algorithm for sperm analysis at the cellular level has a strong potential for improving IVF procedures and their outcomes.

## Supplementary information

Supplementary Information
