# Author Correction: Partially spatially coherent digital holographic microscopy and machine learning for quantitative analysis of human spermatozoa under oxidative stress condition

**DOI:** 10.1038/s41598-019-42490-6

**Published:** 2019-04-10

**Authors:** Vishesh Dubey, Daria Popova, Azeem Ahmad, Ganesh Acharya, Purusotam Basnet, Dalip Singh Mehta, Balpreet Singh Ahluwalia

**Affiliations:** 1 Applied Optics and Biophotonics Laboratory, Department of Physics, Indian Institute of Technology Delhi, Delhi, India; 2 Department of Physics and Technology, UiT The Arctic Univ. of Norway, Tromsø, Norway; 3 Department of Clinical Medicine, UiT The Arctic Univ. of Norway, Tromsø, Norway; 4 Department of Clinical Science, Intervention and Technology Karolinska Univ. Hospital, Karolinska, Sweden

Correction to: *Scientific Reports* 10.1038/s41598-019-39523-5, published online 05 March 2019

This Article contains an error in the order of the Figures. Figures 2 and 3 were published as Figures 3 and 2 respectively. The correct Figures 2 and 3 appear below as Figures 1 and 2. The Figure legends are correct.

**Figure 1:**
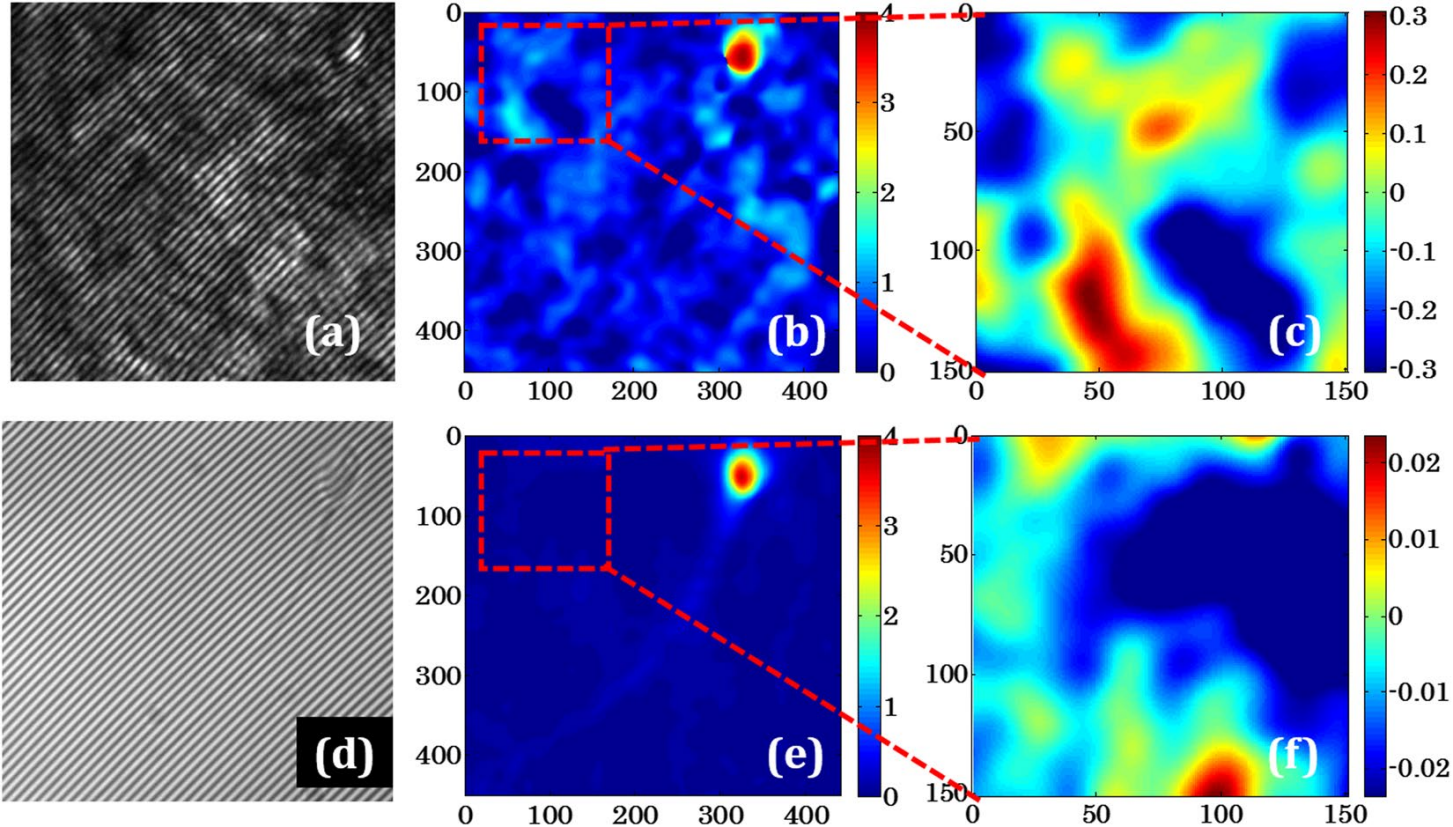
Measurement of the spatial phase sensitivity of QPM for direct laser and pseudo-thermal light sources. (**a**,**d**) are the interferograms obtained with healthy sperm cell as a test specimen, (**b**,**e**) reconstructed phase map of the sperm cell corresponding to (**a**,**d**), respectively and (**c**,**f**) spatial phase noise of the experimental setup for laser and pseudo-thermal light sources, respectively. Note that the scale of the color bars used in (**c**,**f**) having different values.

**Figure 2:**
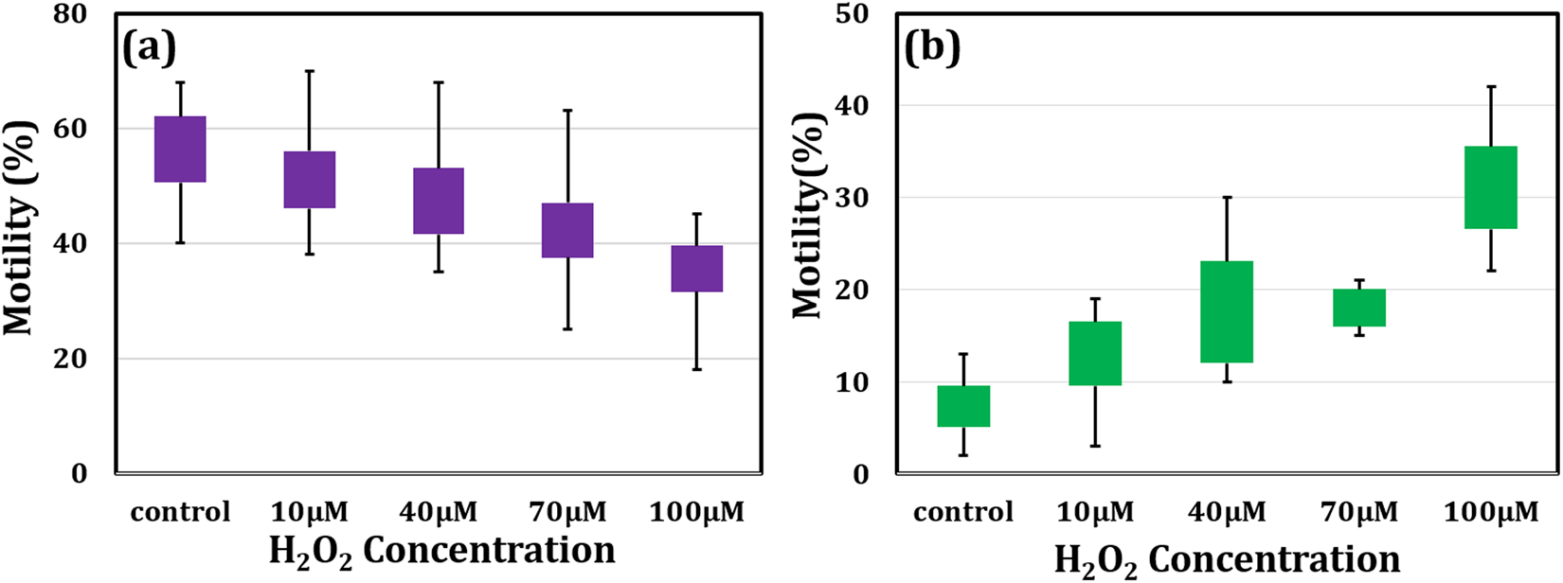
Effects of the H_2_O_2_ on the motility of sperm cells (**a**) changes in the percentage of progressive motility and (**b**) non-progressive motility of sperm cells after H_2_O_2_ treatment at different concentrations comparing to control (mean ± SE, p < 0.05 vs. control).

