# Supplementary material for: Partially spatially coherent digital holographic microscopy and machine learning for quantitative analysis of human spermatozoa under oxidative stress condition
Source: Sci Rep. 2019 Mar 5;9:3564. doi: 10.1038/s41598-019-39523-5 (PMC6401136; doi:10.1038/s41598-019-39523-5)
Supplement: Supplementary file 1 — Supplementary Information [file 41598_2019_39523_MOESM1_ESM.docx]

**Partially spatially coherent digital holographic microscopy and machine learning for quantitative analysis of human spermatozoa under oxidative stress condition**

Vishesh Dubey^1,2,†^, Daria Popova^3,†^, Azeem Ahmad^1,2^, Ganesh Acharya^3,4^, Purusotam Basnet^3^, Dalip Singh Mehta^1^, Balpreet Singh Ahluwalia^2,*^

**Supplementary information**

**Feature extraction for the sperm head**

The phase image of the sperm is extracted from recorded off-axis holograms by writing a Matlab program. The reconstructed phase map of the sperm is further utilized to extract various parameters to describe the morphology of sperm head. Graphical user interface (GUI)[^1^](#_ENREF_1) was developed to isolate the sperm head from the background and tail by putting a threshold value and region of interest (ROI) was selected as shown in fig. 1. The phase image of the sperm cell is used as input of the GUI and the contrast of the image is enhanced for better boundary detection of sperm head. Further, the image was segmented by selecting the boundary and isolation of the sperm head and various morphological and statistical parameters were extracted from it.

A GUI is developed for the automated selection of sperm head (i.e. ROI)[^2^](#_ENREF_2). Following steps are followed to segment the sperm head:

1. A circular/elliptical binary mask is generated to segment sperm head. The circular mask only selects region of the sperm head.
2. The pixel’s phase values of segmented image below the spatial phase noise level (peak to valley noise value) of the system are set to zero to select only ROI containing sperm’s head. This leads to accurate measurement of the phase and subsequently morphological parameters.

**Figure S1**. Graphical user interface developed in Matlab. Visual presentation of the selection steps for the isolation of the sperm head. (a) phase map of whole sperm, (b) selection of circular/elliptical binary mask to isolate sperm head, (c) segmented sperm head and setting the threshold value and (d) reconstructed phase map of isolated sperm head (color bar is in radian).

**Effect of H_2_O_2_ on sperm head**

The effect of oxidative stress generated by H_2_O_2_ has been demonstrated in number of studies[^3-5^](#_ENREF_3). Although in previous studies they measured the effect on the sperm motility, DNA fragmentation etc. but the quantification of biophysical parameters have not done yet. The quantification of the biophysical parameters such as optical thickness, morphology, refractive index distribution can be utilized as a key marker for the selection of healthy sperm in assisted reproductive technology (ART) treatment. The decrease in the optical thickness is observed in the sperm head after introducing H_2_O_2_ as shown in Fig. S2. The structure of sperm cell suggest that maximum change in phase occurs where the nucleus is located as shown in the Fig. 4 of the revised manuscript. The results also supports the findings reported earlier in the literature[^6-8^](#_ENREF_6). This change in maximum phase at a particular location of the sperm head indicates the deformation/flattening of the nucleus of the sperm. Since our technique is label-free, we are not capable to detect the boundaries of the nucleus. According to literature, the origin of decrease in the maximum phase of sperm head could be due to various possible reasons such as: deformation in nucleus, structural organization of sperm DNA etc[^6^](#_ENREF_6)^,^[^9^](#_ENREF_9). The morphometric values obtained in our study can provide the volumetric estimation for the quantitative comparison between control and H_2_O_2_ treated sperm cells. The correlation of decrease in the phase and deformation in the nucleus can be quantify by multimodal imaging in future where the boundary can be located by fluorescence imaging and QPM can provide the changes in the maximum phase of nucleus[^8^](#_ENREF_8).

**Figure S2.** Schematic diagram of a sperm cell. The morphological changes occurs in sperm head during oxidative stress. The decrease in the optical thickness is observed in the sperm head after introducing H_2_O_2_.

**Support Vector Machine**

Support vector machine (SVM) is the one of the most popular machine learning techniques[^10-12^](#_ENREF_10). It is a supervised learning model mostly applied for the classification and regression analysis[^10^](#_ENREF_10)^,^[^12^](#_ENREF_12). Here, we have employed SVM for the binary classification of the state of the specimen[^10^](#_ENREF_10)^,^[^13^](#_ENREF_13). The samples are divided into two different set of data i.e. training and testing data[^12^](#_ENREF_12). If we have n number of features then the data is plotted in n dimensional space for the classification. In the n dimensional space a linear surface is constructed and the special properties of this decision surface insures maximum classification ability of the learning algorithm. In 2D space this linear surface transformed into a line namely linear hyperplane. The approach of SVM may roughly stated as follow[^12^](#_ENREF_12)^,^[^14^](#_ENREF_14):

**Class separation:** For the classification of the input data in to two different classes suppose C_1_ and C_2_ by drawing a hyperplane line with maximum margin between the data points of different groups. The points on the margin boundaries of the hyperplane are called support vectors and the wider margin prevents the misclassification of the data points.

**Figure S2**. A separable classification by support vector algorithm. Geometric interpretation of the soft margin SVM in a two-dimensional feature space.

**Overlapping classes:** The data points present on the wrong side of the hyperplane and margin are treated in such a way that it does not influence the results i.e. they are weighted down. This separation is called soft margin.

**Non-linear hyperplane:** When the data points are messy and the linear separator cannot be found then the data points are projected in higher dimensional space where the data sets effectively became linearly separable. The kernel techniques are widely used for the realization of the projection in higher dimensional space.

The whole task of the classification can be formulated as a quadratic optimization problem. Although, it looks as a linear algorithm in high-dimensional space, it does not require any computations in that high-dimension space. All the calculations are directly performed in input space by the use of the kernels. This is the key advantage of the SVM that it deals with complex algorithms for non-linear pattern recognition or feature extraction.

From fig. S2 it is observed that we account only a small section of data points called the support vectors to construct the optimal hyperplane. For the supervised learning we have train the program by providing the inputs and results of classes C_1_ and C_2_.

For the linear hyperplane case, the optimal hyperplane can be represented as[^12^](#_ENREF_12)^,^[^15^](#_ENREF_15)

$$w\cdot x_{i}+b=0 (S1)$$

The classification of the data points is performed in such a way that it fulfills the condition:

$$w\cdot x_{i}+b>0 \to x_{i}\in C_{1} (S2)$$

$$and <0 \to x_{i}\in C_{2}$$

To design the classifier, we know the state of training data $x_{i}$ belongs to either class C_1_ or class C_2_ and hence with each data point we can assign a class belongs to it. So along with each $x_{i}$we can put a $y_{i}$ in such a way that $y_{i}$ is either +1 or -1 for class C_1_ and C_2_, respectively. Further if we compute the product $y_{i}\left( w\cdot x_{i}+b \right),$ it will always be greater than zero for all the classes.

The distance of a data point *x* from the hyperplane in eq. S1 can be written as[^11^](#_ENREF_11)^,^[^14^](#_ENREF_14)

$$\frac{w\cdot x+b}{\left\| \left. w \right\| \right.}\geq\gamma(S3)$$

or $w.x+b\geq\gamma\cdot\left\| \left. w \right\| \right.$

where γ is the margin of the *x* from the hyperplane and $\gamma\cdot\left\| \left. w \right\| \right.$ can be scale to unity for simplicity.

Further, for the fulfillment of the condition of support vectors the eq. S2 can be rewrite as:

$$w\cdot x_{i}+b>1 \to x_{i}\in C_{1} (S4)$$

$$and <1 \to x_{i}\in C_{2}$$

To maximize the margin γ, we have to minimize the weight vector *w* and by solving the Lagrangian we find the expression of *w* as[^16^](#_ENREF_16):

$$w=\sum_{i=1}^{m} \alpha_{i}y_{i}x_{i} (S5)$$

Where *α_i_* is the Lagrangian multiplier. Once we have the values of *w* and *b* from training data, we can perform the classification of an unknown data using SVM algorithm.

For a unknown data z, the decision *D(z)* can be expressed as[^11^](#_ENREF_11)^,^[^16^](#_ENREF_16):

$$D\left( z \right)=sgn\left( \left. \sum_{j=1}^{m} \alpha_{j}y_{j}x_{j}\cdot z+b \right) (S6) \right.$$

For the classification of the classes C_1_ and C_2_, we don’t need to compute the value of the *D(z)*, we only required the sign of the function. If it is positive then the unknown data belongs to class C_1_ and if sign is negative then it belongs to class C_2_.

**Receiver Operating Characteristic curve**

For most of the diagnostic tests, a threshold value on the original scale is selected as decision cut off to define negative or positive test outcome. Receiver Operative Characteristic (ROC) curves are widely utilized to describe and compare the performance and accuracy of the classifier[^17-19^](#_ENREF_17). The ROC plot is obtained by measuring sensitivity and specificity of the every observed data and plotting sensitivity against 1-specificity as shown in Fig. S3[^20^](#_ENREF_20). If the plot is a straight line from the bottom of the left corner to the top of the right corner as shown in Fig. S3, the test is completely useless[^21^](#_ENREF_21). In general, there is always some overlap of the values in two classes so the curve lie somewhere in the upper left half extreme and the area under the curve (AUC) decides the accuracy of the classifier[^18^](#_ENREF_18)^,^[^21^](#_ENREF_21).

**Figure S3**. ROC curve for testing dataset of sperm head for control and 10μM/ml H_2_O_2_ concentration treated sperm cells using various parameters.

The sensitivity or true positive rate (TPR) of the classifier is defined as conditional probability of correctly identification of the disease by the classifier and given as[^19^](#_ENREF_19)^,^[^20^](#_ENREF_20):

$$Sensitivity =\frac{True positive}{True positive+False negative}$$

The true negative rate (TNR) or specificity is defined as the conditional probability of the correct labelling of the non-disease subject by the test and expressed as[^19^](#_ENREF_19):

$$Specificity = \frac{True negative}{True negative+False positive}$$

 The false positive rate (FPR) or 1-specificity is defined as the probability of the conditional positive for non-disease subjects and expressed as the ratio of the false positive to the sum of true negative and false positive[^19^](#_ENREF_19)^,^[^20^](#_ENREF_20):

$$1-Specificity or False positive rate(FPR) = \frac{False positive}{True negative+False positive}$$

From past few decades, the ROC analysis has become a popular method to evaluate the accuracy of the systems especially in medical applications. The area under curve (AUC) is one of the most important indices of the ROC to measure the accuracy of the classifier. If the AUC having maximum value=1 that means the classifier is perfect and the diagnostic test is 100% able to differentiate between the diseased and non-diseased. The specificity and sensitivity are evaluated as the measure of accuracy of the diagnostic test and the accuracy of the classifier is defined as[^19^](#_ENREF_19)^,^[^20^](#_ENREF_20):

$$Accuracy (ACC) = \frac{True positive+True negative}{Total population}$$

**References**

1 https://in.mathworks.com/help/matlab/creating_guis/about-the-simple-guide-gui-example.html;jsessionid=d81bcb018e24231836c8ef1de556.

2 https://in.mathworks.com/help/images/roi-based-processing.html.

3 Whittington, K. et al. Reactive oxygen species (ROS) production and the outcome of diagnostic tests of sperm function. International journal of andrology 22, 236-242 (1999).

4 Kao, S.-H. et al. Increase of oxidative stress in human sperm with lower motility. Fertility and sterility 89, 1183-1190 (2008).

5 Duru, N. K., Morshedi, M. & Oehninger, S. Effects of hydrogen peroxide on DNA and plasma membrane integrity of human spermatozoa. Fertility and sterility 74, 1200-1207 (2000).

6 Di Caprio, G. et al. Holographic imaging of unlabelled sperm cells for semen analysis: a review. Journal of biophotonics 8, 779-789 (2015).

7 Mirsky, S., Barnea, I. & Shaked, N. Label-Free quantitative imaging of sperm for in vitro fertilization using interferometric phase microscopy. J Fertil In Vitro-IVF-Worldwide Reprod Med Genet Stem Cell Biol 190 (2016).

8 Haifler, M. et al. Interferometric phase microscopy for label-free morphological evaluation of sperm cells. Fertility and sterility 104, 43-47. e42 (2015).

9 De Iuliis, G. N. et al. DNA damage in human spermatozoa is highly correlated with the efficiency of chromatin remodeling and the formation of 8-hydroxy-2′-deoxyguanosine, a marker of oxidative stress. Biology of reproduction 81, 517-524 (2009).

10 Hearst, M. A., Dumais, S. T., Osuna, E., Platt, J. & Scholkopf, B. Support vector machines. IEEE Intelligent Systems and their applications 13, 18-28 (1998).

11 Ganapathiraju, A. Support Vector Machines-A First Look. Institute for Signal and Information Processing (1998).

12 Steinwart, I. & Christmann, A. Support vector machines. (Springer Science & Business Media, 2008).

13 Wernick, M. N., Yang, Y., Brankov, J. G., Yourganov, G. & Strother, S. C. Machine learning in medical imaging. IEEE signal processing magazine 27, 25-38 (2010).

14 Schölkopf, B., Bartlett, P., Smola, A. & Williamson, R. in ICANN 98 111-116 (Springer, 1998).

15 Boser, B. E., Guyon, I. M. & Vapnik, V. N. in Proceedings of the fifth annual workshop on Computational learning theory. 144-152 (ACM).

16 Bottou, L., Chapelle, O., Decoste, D. & Weston, J. Large-scale kernel machines. (MIT press, 2007).

17 Zweig, M. H. & Campbell, G. Receiver-operating characteristic (ROC) plots: a fundamental evaluation tool in clinical medicine. Clinical chemistry 39, 561-577 (1993).

18 Hanley, J. A. & McNeil, B. J. A method of comparing the areas under receiver operating characteristic curves derived from the same cases. Radiology 148, 839-843 (1983).

19 Greiner, M., Pfeiffer, D. & Smith, R. Principles and practical application of the receiver-operating characteristic analysis for diagnostic tests. Preventive veterinary medicine 45, 23-41 (2000).

20 Schafer, H. Efficient confidence bounds for ROC curves. Statistics in medicine 13, 1551-1561 (1994).

21 Altman, D. G. & Bland, J. M. Diagnostic tests 3: receiver operating characteristic plots. BMJ: British Medical Journal 309, 188 (1994).
